# BMI-specific nonlinear associations and threshold effects of the atherogenic index of plasma on incident prediabetes: insights from 100473 Chinese adults

**DOI:** 10.3389/fendo.2026.1813692

**Published:** 2026-05-14

**Authors:** Bing Wang, Weiqin Shen, Baoyin Li, Yesheng Pan

**Affiliations:** Department of Cardiology, Jinshan Branch of Shanghai Sixth People’s Hospital, Shanghai, China

**Keywords:** atherogenic index of plasma, body mass index, Chinese adults, interaction, nonlinear association, prediabetes, risk stratification

## Abstract

**Background:**

Emerging evidence suggests that the atherogenic index of plasma (AIP), a simple marker of lipid-related metabolic risk, may relate to dysglycemia in a nonlinear fashion. However, whether this association differs across body mass index (BMI) categories, particularly in terms of nonlinear risk patterns and thresholds, remains unclear.

**Methods:**

We analyzed data from 100,473 Chinese adults who underwent baseline health examinations and had baseline FPG < 5.6 mmol/L. Because HbA1c and OGTT data were unavailable, the outcome was defined as FPG-defined incident prediabetes (5.6-6.9 mmol/L). Participants were classified into underweight, normal weight, overweight, and obesity groups according to Chinese BMI criteria. Restricted cubic spline (RCS) and Cox regression were used to evaluate BMI-specific associations between AIP and prediabetes risk and to test for interaction by BMI.

**Results:**

During a median follow-up of 3.0 years, 12,371 participants developed FPG-defined incident prediabetes. A significant interaction between AIP and BMI was observed (P for interaction < 0.001). BMI-stratified analyses showed that AIP was associated with FPG-defined prediabetes risk only among normal-weight and overweight participants, but not among underweight or obese participants. RCS analyses further identified nonlinear associations in these two BMI groups, with model-derived inflection points at AIP = -0.20 in normal-weight individuals and AIP = 0.057 in overweight individuals.

**Conclusion:**

BMI modified the association between AIP and FPG-defined prediabetes risk. Significant nonlinear and threshold-dependent associations were observed only in normal-weight and overweight Chinese adults.

## Introduction

Prediabetes is a reversible intermediate state between normoglycemia and type 2 diabetes mellitus (T2DM), associated with a substantially increased risk of diabetes and cardiovascular disease (1–3). Worldwide, an estimated 536 million individuals had diabetes in 2021, and the number of people with prediabetes is projected to exceed 587 million by 2030 (4). In China, national surveys indicate an age-adjusted prediabetes prevalence of approximately 35%, affecting more than one-third of Chinese adults (5). These trends underscore the importance of systematically assessing key risk factors that contribute to the development of prediabetes.

The atherogenic index of plasma (AIP), calculated as the logarithm of the ratio of triglyceride to high-density lipoprotein cholesterol, reflects the degree of dyslipidemia and lipid metabolism imbalance in the body (6). Current evidence suggests that the relationship between AIP and glycemic abnormalities may be nonlinear, implying that even modest increases in AIP within a certain range could disproportionately elevate diabetes risk (7–9). However, the factors that influence this nonlinear association remain largely unexplored. Body mass index (BMI), a widely used indicator of adiposity, profoundly affects insulin sensitivity, lipid handling, and systemic inflammation (10, 11). Although obesity is a well-established risk factor for dysglycemia, dyslipidemia-related metabolic dysfunction may also play a crucial role among individuals with normal weight (12). In addition, in lean or underweight individuals, lipid metabolism disorders may coexist with a ‘hidden metabolic obesity’ phenotype characterized by insulin resistance and ectopic fat accumulation, which may predispose them to dysglycemia. Conversely, in those with obesity, excessive adiposity may attenuate or mask the contribution of lipid imbalance to glucose dysregulation (13, 14). Nevertheless, no large-scale prospective cohort study has systematically evaluated whether BMI modifies the nonlinear association between AIP and prediabetes risk. Therefore, using data from a large cohort of 100,473 Chinese adults undergoing health examinations, we aimed to investigate whether BMI modifies the nonlinear relationship between AIP and the risk of prediabetes.

## Methods

### Data source

This study was a secondary analysis of the dataset collected by Chen et al., which included 211,833 participants and is publicly available in the Dryad Repository (15). The original study was approved by the Rich Healthcare Group Review Board, and the requirement for informed consent was waived because of its retrospective design. The original study was conducted in accordance with the Declaration of Helsinki. Because the present analysis used only fully de-identified and publicly available data, no additional institutional review board approval or informed consent was required.

**Figure 1:**
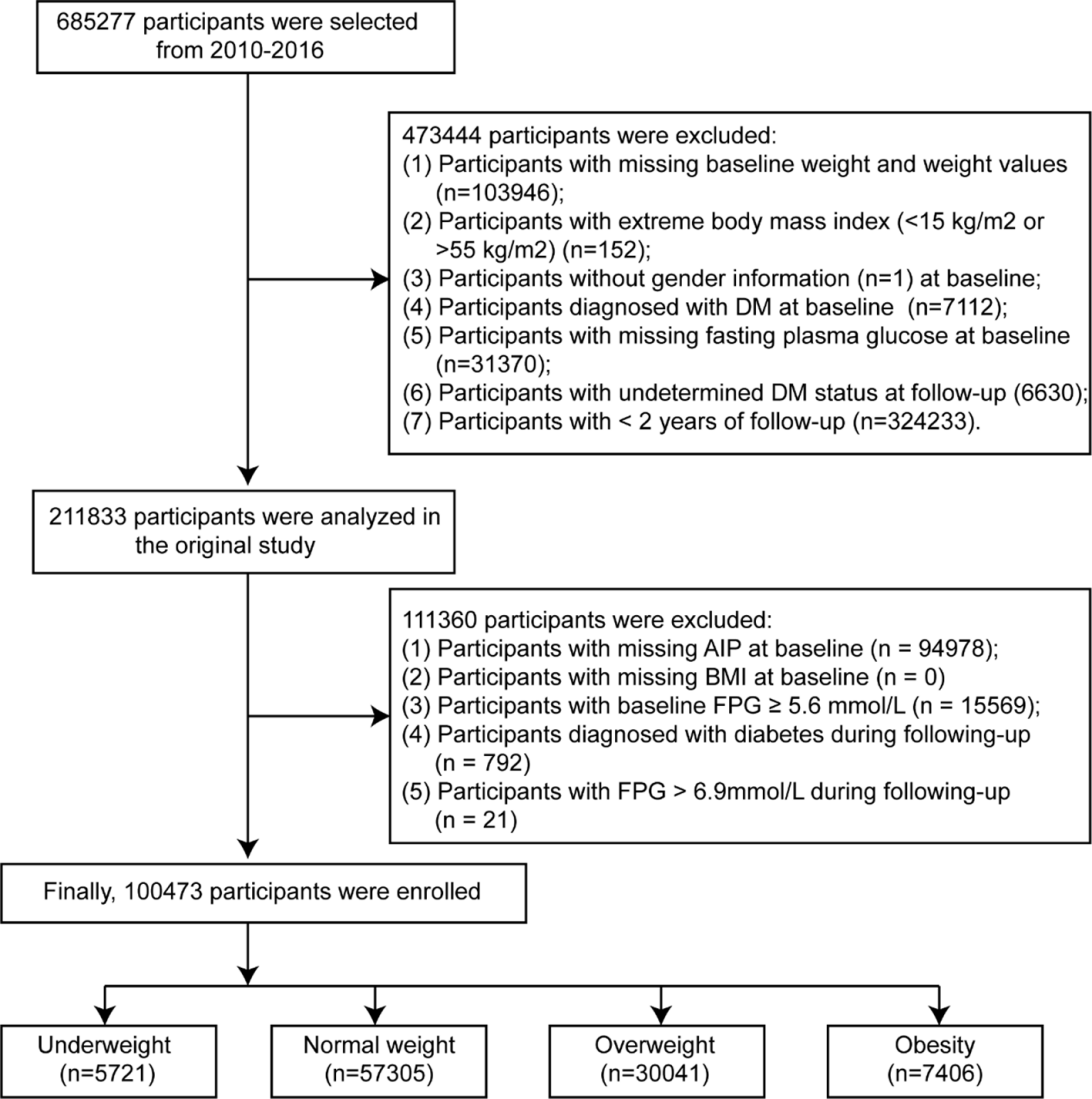
The flowchart. BMI body mass index, FPG fasting plasma glucose, AIP atherogenic index of plasma.

Based on the objectives of our study, we applied additional exclusion criteria to the original dataset to construct a normoglycemic cohort for assessing prediabetes risk. Individuals were excluded if they had missing AIP measurements (n = 94978), missing BMI data (n = 0), baseline fasting plasma glucose (FPG) ≥ 5.6 mmol/L (n = 15569), a diagnosis of diabetes during follow-up (n = 792), or FPG > 6.9 mmol/L during follow-up (n = 21). The final analytical sample comprised 100473 participants. Based on BMI standards for the Chinese population (16), all participants were categorized into four groups: underweight (n = 5721), normal weight (n = 57305), overweight (n = 30041), and obesity (n = 7406) (**Figure 1**).

### Definition of AIP

AIP was calculated from baseline triglyceride (TG) and baseline high-density lipoprotein cholesterol (HDL-C) and served as an indicator of atherogenic dyslipidemia (17). We also performed an exploratory discrimination analysis to assess whether AIP may provide supplementary, partially overlapping information beyond triglyceride-glucose (TyG) index. The TyG index was derived from baseline TG and baseline FPG (18). These indices were computed using the following formulas: AIP = log_10_(TG/HDL) and TyG index = ln (TG*FPG/2).

### Definition of prediabetes

Hemoglobin A1c (HbA1c) and 2-hour plasma glucose during oral glucose tolerance testing (OGTT) were not available in the source dataset; therefore, incident prediabetes in the present study was defined solely according to FPG. In accordance with the FPG criterion of the 2018 ADA standards, FPG-defined incident prediabetes was defined as an FPG level of 5.6-6.9 mmol/L during follow-up among individuals with baseline FPG < 5.6 mmol/L who did not progress to diabetes (19). Because HbA1c and OGTT data were unavailable, individuals with isolated HbA1c-defined prediabetes or impaired glucose tolerance could not be identified. Therefore, the outcome in this study should be interpreted as FPG-defined incident prediabetes rather than prediabetes defined by the full ADA criteria.

### Covariates collection

Baseline information on metabolic and clinical characteristics was collected, including demographic variables (age and sex), blood pressure indices (systolic blood pressure [SBP] and diastolic blood pressure [DBP]), lifestyle factors (smoking status and drinking status), family history of diabetes, lipid profiles (total cholesterol [TC], TG, HDL-C, and low-density lipoprotein cholesterol [LDL-C]), liver enzymes (alanine aminotransferase [ALT] and aspartate aminotransferase [AST]), renal function markers (blood urea nitrogen [BUN] and creatinine [Scr]), FPG, and BMI, which was calculated as weight (kg) divided by height squared (m²).

To minimize potential bias and loss of statistical power, missing covariate data were handled using multiple imputation by chained equations (MICE), generating ten imputed datasets. The imputation model included the exposure (AIP), FPG-defined incident prediabetes, follow-up time, and all covariates used in the multivariable analyses. FPG-defined incident prediabetes and follow-up time were included as predictors but were not themselves imputed. Missing values in AIP were not imputed because AIP was the primary exposure variable, and imputing the main exposure may introduce model-dependent error and potential exposure misclassification; therefore, participants with missing AIP were excluded from the analytic sample. To examine the potential impact of this exclusion, we compared baseline characteristics between participants included in the analytic cohort and those excluded because of missing AIP or other exclusion criteria. Estimates across the imputed datasets were combined using Rubin’s rules (20). The proportions of missingness for each variable are presented in **Supplementary Table 1**. To assess the plausibility of the imputed data, we descriptively compared the distributions of key variables before and after multiple imputation; overall, no material differences were observed (**Supplementary Table 2**). The primary analyses were conducted using the imputed dataset.

### Statistical analysis

Statistical analyses were performed using R software (version 4.2.2) and STATA software (version 15.0). Baseline characteristics were summarized across BMI categories, with continuous variables presented as means ± standard deviations or medians (interquartile ranges) and categorical variables as percentages. Differences among BMI groups were assessed using one-way ANOVA or the Kruskal-Wallis test for continuous variables and the χ² test for categorical variables.

Prior to model fitting, multicollinearity among covariates was evaluated using variance inflation factors (VIFs), and all variables showed acceptable VIF values (< 5) (**Supplementary Table 3**), indicating no evidence of collinearity. Cox proportional hazards regression was used to quantify the association between AIP and prediabetes risk, expressed as hazard ratios (HRs) with 95% confidence intervals (CIs). Three hierarchical models were constructed: Model 1 adjusted for age and sex; Model 2 further adjusted for SBP, DBP, smoking status, drinking status, and family history of diabetes; and Model 3 additionally adjusted for TC, FPG, LDL-C, ALT, AST, BUN, and Scr. AIP was evaluated both as a continuous variable and in tertiles, with tests for trend performed by assigning the median value of each tertile. For Kaplan-Meier analyses, AIP tertiles were defined separately within each BMI stratum to allow balanced within-stratum comparisons of cumulative prediabetes incidence. Restricted cubic spline (RCS) models with four knots placed at the 5th, 35th, 65th, and 95th percentiles were used to explore potential nonlinear associations between AIP and prediabetes. When a nonlinear association was suggested by the RCS analysis, additional threshold-effect analyses were performed using fully adjusted two-piece Cox proportional hazards models within each BMI stratum. The estimated turning point was identified using a bootstrap-based likelihood search and was defined as the value yielding the maximum log-likelihood. Effect modification by BMI categories was assessed by introducing multiplicative interaction terms between AIP and BMI into the fully adjusted model, with significance tested using likelihood ratio tests.

Time-dependent receiver operating characteristic (ROC) analyses were conducted in these two BMI subgroups. The predictive performance of AIP, the TyG index, and their combined model was assessed at 3-, 4-, and 5-year follow-up using area under the curve (AUC) values, and supplementary discriminatory information of AIP was explored by comparing AUCs across models. Additional subgroup analyses were performed by age (≤60/>60 years old), sex (Male/Female), and family history of diabetes (Yes/No). Statistical significance was defined as a two-sided *P* < 0.05.

## Results

### Baseline characteristics of participants

A total of 100473 participants were included in the present analysis, with a mean age of 42.91 ± 12.46 years; 51.97% were men and 48.03% were women. **Table 1** summarizes the baseline characteristics of participants according to BMI categories [underweight (<18.5 kg/m²), normal weight (18.5-23.9 kg/m²), overweight (24.0-27.9 kg/m²), and obese (≥28 kg/m²)]. A total of 100473 participants were included, with 5721 (5.7%) underweight, 57305 (57.0%) normal weight, 30041 (29.9%) overweight, and 7406 (7.4%) obesity. The prediabetes incidence increased markedly with BMI, from 5.0% in underweight to 21.5% in obesity (*P* < 0.001). BMI was positively associated with age, blood pressure, and most cardiometabolic parameters (all *P* < 0.001). Participants with higher BMI were older and more often male. Mean SBP and DBP increased progressively across BMI groups. Higher-BMI individuals were also more likely to be current smokers and drinkers (both *P* < 0.001). FPG, TC, TG, LDL-C, ALT, AST, BUN, and Scr all increased significantly, whereas HDL-C decreased (all *P* < 0.001). The median AIP rose steadily from -0.32 in the underweight group to 0.13 in the obese group. Family history of diabetes did not differ significantly among groups (*P* = 0.09).

**Table 1:** Baseline characteristics of participants by BMI categories (underweight, normal weight, overweight, and obesity).

Variables	Underweight (n=5721)	Normal weight (n=57305)	Overweight (n=30041)	Obesity (n=7406)	*P*
Age, years	36.55 ± 10.93	41.86 ± 12.04	45.74 ± 12.69	44.52 ± 12.77	<0.001
Sex, n (%)					<0.001
Male	1435(25.08)	24322(42.44)	20905(69.59)	5555(75.01)	
Female	4286(74.92)	32983(57.56)	9136(30.41)	1851(24.99)	
SBP, mmHg	109.25 ± 13.42	114.93 ± 14.99	123.07 ± 15.74	129.01 ± 16.37	<0.001
DBP, mmHg	68.99 ± 9.08	71.61 ± 9.93	76.99 ± 10.73	80.87 ± 11.55	<0.001
BMI	17.61 ± 0.70	21.47 ± 1.49	25.58 ± 1.09	29.87 ± 1.88	<0.001
Smoking status, n (%)					<0.001
Current	678(11.85)	8835(15.42)	6785(22.59)	1890(25.52)	
Once	134(2.34)	1933(3.37)	1376(4.58)	347(4.69)	
Never	4909(85.81)	46537(81.21)	21880(72.83)	5169(69.79)	
Drinking status, n (%)					<0.001
Current	68(1.19)	1039(1.81)	808(2.69)	237(3.20)	
Once	620(10.84)	7689(13.42)	5687(18.93)	1473(19.89)	
Never	5033(87.97)	48577(84.77)	23546(78.38)	5696(76.91)	
Family history of diabetes, n (%)					0.09
No	5615(98.15)	55999(97.72)	29410(97.90)	7237(97.72)	
Yes	106(1.85)	1306(2.28)	631(2.10)	169(2.28)	
FPG, mmol/L	4.66 ± 0.48	4.76 ± 0.47	4.85 ± 0.46	4.88 ± 0.47	<0.001
TC, mmol/L	4.47 ± 0.82	4.67 ± 0.86	4.89 ± 0.89	5.00 ± 0.89	<0.001
TG, mmol/L	0.73(0.58,0.97)	0.92(0.68,1.30)	1.37(0.97,2.00)	1.66(1.20,2.36)	<0.001
HDL-C, mmol/L	1.55 ± 0.32	1.43 ± 0.30	1.29 ± 0.28	1.25 ± 0.27	<0.001
LDL-C, mmol/L	2.51 ± 0.61	2.69 ± 0.66	2.85 ± 0.68	2.91 ± 0.68	<0.001
ALT, U/L	12.80(10.00,16.30)	15.30(11.70,21.80)	22.80(16.00,33.10)	30.40(20.60,47.00)	<0.001
AST, U/L	19.66(16.00,24.04)	21.00(16.85,26.00)	24.00(19.00,30.20)	27.10(21.10,35.60)	<0.001
BUN, mmol/L	4.35 ± 1.12	4.55 ± 1.15	4.81 ± 1.16	4.82 ± 1.12	<0.001
Scr, umol/L	62.54 ± 13.02	67.54 ± 15.21	74.44 ± 15.73	75.42 ± 15.06	<0.001
AIP	-0.32(-0.44,-0.18)	-0.19(-0.35,0.00)	0.03(-0.15,0.23)	0.13(-0.03,0.31)	<0.001
Prediabetes, n (%)					<0.001
No	5433(94.97)	51902(90.57)	24952(83.06)	5815(78.52)	
Yes	288(5.03)	5403(9.43)	5089(16.94)	1591(21.48)	

SBP, systolic blood pressure; DBP, diastolic blood pressure; BMI, body mass index; FPG, fasting plasma glucose; TC, total cholesterol; TG, triglyceride; HDL-C, high-density lipoprotein cholesterol; LDL-C, low-density lipoprotein cholesterol; ALT, alanine aminotransferase; AST, aspartate aminotransferase; BUN, blood urea nitrogen; Scr, serum creatinine; AIP, atherogenic index of plasma.

In addition, the baseline characteristics of participants with and without prediabetes are presented in **Supplementary Table 4**. Compared with participants without prediabetes, those who developed prediabetes were older and more likely to be men. Individuals with prediabetes had higher mean systolic and diastolic blood pressure, BMI, and fasting plasma glucose levels (all *P* < 0.001). Moreover, participants with prediabetes exhibited a more adverse lipid profile. Liver and renal function indices were also elevated in the prediabetes group (all *P* < 0.001). The median AIP was higher among participants with prediabetes compared with those without (-0.003 vs. -0.12; *P* < 0.001). In addition, compared with the included participants, the excluded participants differed significantly in multiple baseline characteristics and generally exhibited a less favorable metabolic profile (**Supplementary Table 5**).

### BMI-stratified associations between AIP and prediabetes risk

**Figure 2:**
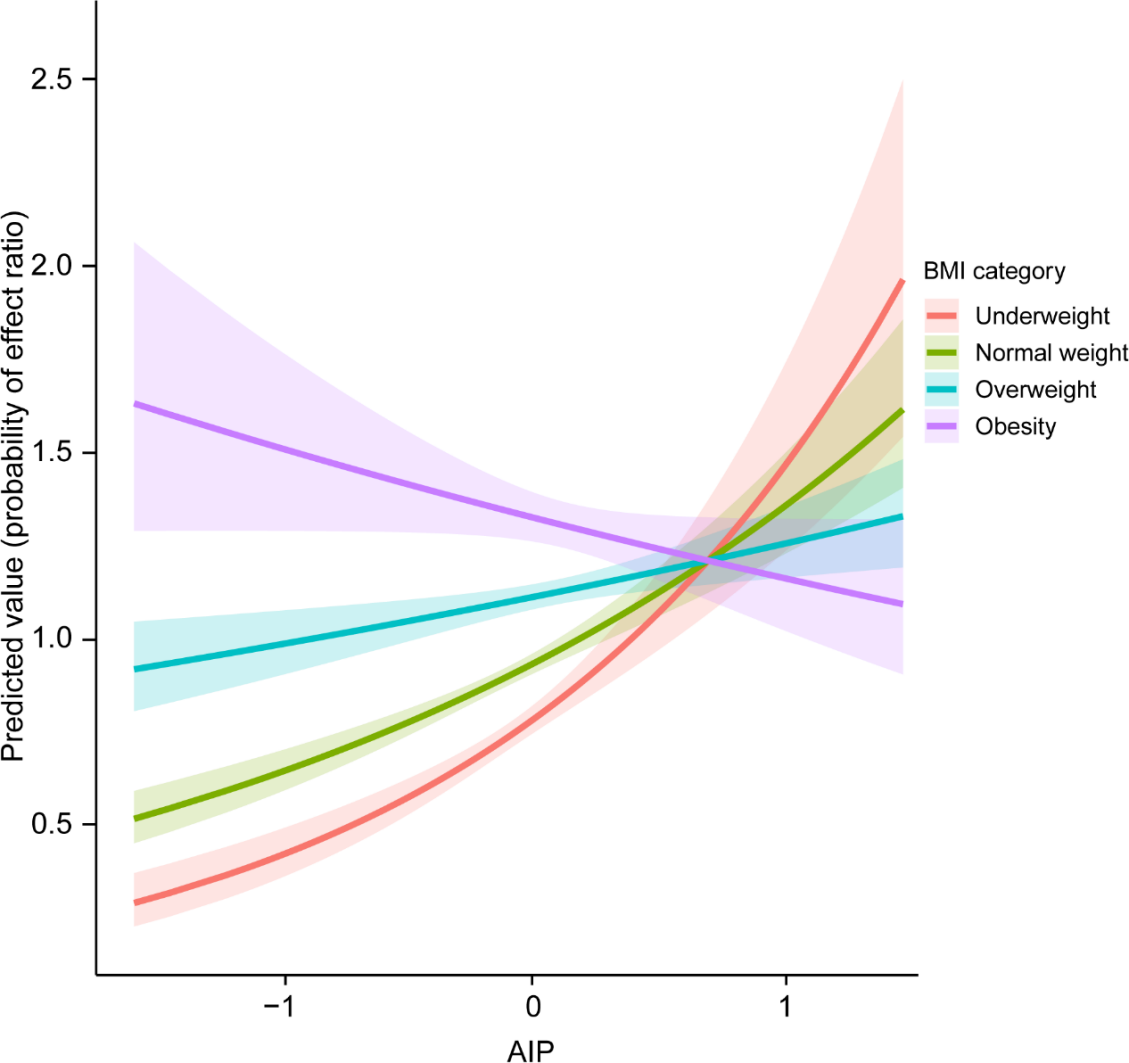
Interaction plot of BMI categories and AIP on prediabetes risk. The model was adjusted for age, sex, SBP, DBP, smoking status, drinking status, family history of diabetes, TC, FPG, LDL-C, ALT, AST, BUN, and Scr. SBP systolic blood pressure, DBP diastolic blood pressure, BMI body mass index, FPG fasting plasma glucose, TC total cholesterol, TG triglyceride, HDL-C high-density lipoprotein cholesterol, LDL-C low-density lipoprotein cholesterol, ALT alanine aminotransferase, AST aspartate aminotransferase, BUN blood urea nitrogen, Scr serum creatinine, AIP atherogenic index of plasma.

**Figure 3:**
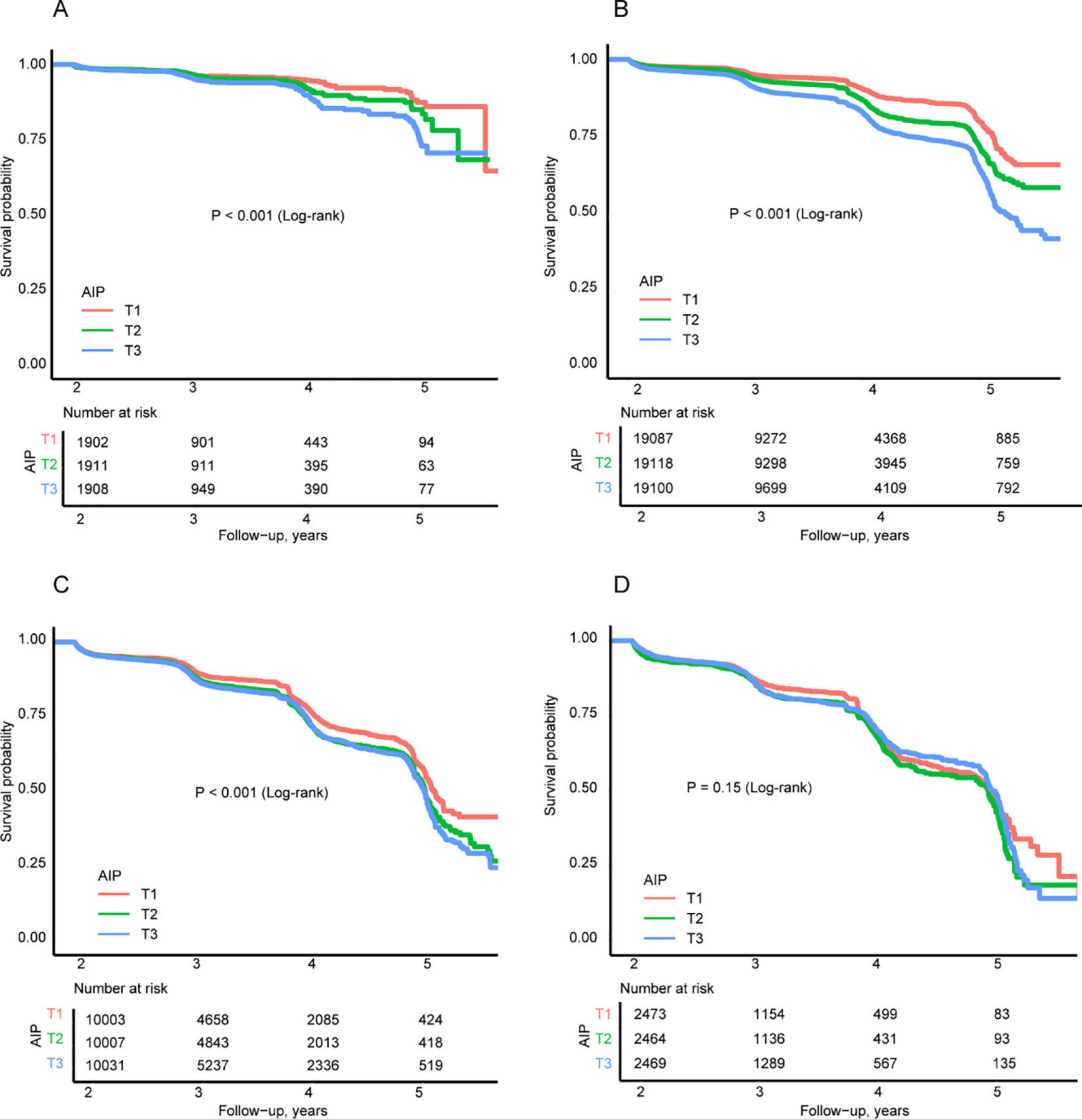
Kaplan-Meier curves for FPG-defined incident prediabetes according to BMI-stratum-specific tertiles of AIP. **(A)**, Underweight; **(B)**, Normal weight; **(C)**, Overweight; and **(D)**, Obesity. Within each BMI category, participants were divided into tertiles according to the distribution of AIP in that specific stratum. BMI body mass index, AIP atherogenic index of plasma; FPG, fasting plasma glucose.

During a median follow-up of 3.0 years, 12,371 participants developed FPG-defined incident prediabetes. In the overall population, higher AIP was significantly associated with an increased risk of prediabetes in a nonlinear manner (**Supplementary Table 6** and **Supplementary Figure 1**). Importantly, a significant interaction between AIP and BMI was observed (*P* for interaction < 0.001), indicating that BMI modified the association between AIP and glycemic deterioration (**Figure 2**). Accordingly, BMI-stratified analyses were performed to further characterize this heterogeneity. **Figure 3** presented the Kaplan-Meier survival curves for incident prediabetes according to BMI-stratum-specific tertiles of AIP. Across underweight, normal-weight, and overweight individuals (**Figures 3A–C**), participants with higher AIP values had a significantly higher cumulative incidence of prediabetes (log-rank *P* < 0.001). The separation of survival curves was most evident in normal-weight individuals, whereas the difference was not significant among those with obesity (**Figure 3D**). **Table 2** shows the multivariable-adjusted HRs for prediabetes associated with AIP. Among normal-weight participants, each one-unit increase in continuous AIP was associated with a 36% higher risk of prediabetes in the fully adjusted model (HR = 1.36, 95% CI 1.23-1.51, *P* < 0.001), and those in the highest AIP tertile (T3) had a 20% greater risk compared with the lowest tertile (T1). In overweight individuals, the association remained but was attenuated (HR = 1.14, 95% CI 1.02-1.27). However, no significant association was observed in underweight or obese participants after multivariable adjustment (*P* > 0.05).

**Table 2:** Associations between the AIP and prediabetes stratified by BMI categories (underweight, normal weight, overweight, and obesity).

Groups	Model 1		Model 2		Model 3	
	HR (95%CI)	*P*	HR (95%CI)	*P*	HR (95%CI)	*P*
Underweight						
AIP (Continuous)	2.25(1.32,3.84)	0.003	2.06(1.21,3.49)	0.01	1.33(0.78,2.28)	0.3
The tertiles of AIP						
T1	Reference		Reference		Reference	
T2	1.23(0.96,1.59)	0.11	1.18(0.92,1.53)	0.2	0.99(0.77,1.29)	0.96
T3	1.46(0.96,2.22)	0.08	1.39(0.91,2.12)	0.12	1.12(0.73,1.71)	0.61
*P* for trend		0.03		0.07		0.74
Normal weight						
AIP (Continuous)	1.72(1.56,1.91)	< 0.001	1.61(1.46,1.79)	< 0.001	1.36(1.23,1.51)	< 0.001
The tertiles of AIP						
T1	Reference		Reference		Reference	
T2	1.21(1.14,1.30)	< 0.001	1.19(1.11,1.27)	< 0.001	1.13(1.06,1.21)	< 0.001
T3	1.40(1.30,1.50)	< 0.001	1.34(1.24,1.43)	< 0.001	1.20(1.11,1.29)	< 0.001
*P* for trend		< 0.001		< 0.001		< 0.001
Overweight						
AIP (Continuous)	1.31(1.18,1.44)	< 0.001	1.25(1.13,1.38)	< 0.001	1.14(1.02,1.27)	0.02
The tertiles of AIP						
T1	Reference		Reference		Reference	
T2	1.12(1.02,1.23)	0.02	1.11(1.01,1.22)	0.03	1.08(0.98,1.18)	0.13
T3	1.27(1.16,1.38)	< 0.001	1.23(1.13,1.34)	< 0.001	1.14(1.05,1.25)	0.003
*P* for trend		< 0.001		< 0.001		0.002
Obesity						
AIP (Continuous)	1(0.84,1.20)	0.97	0.97(0.80,1.16)	0.72	0.99(0.81,1.21)	0.92
The tertiles of AIP						
T1	Reference		Reference		Reference	
T2	1.06(0.85,1.31)	0.62	1.04(0.84,1.30)	0.69	1.04(0.84,1.30)	0.7
T3	1.10(0.90,1.35)	0.34	1.08(0.88,1.32)	0.45	1.10(0.90,1.35)	0.36
*P* for trend		0.27		0.38		0.25

Model 1 adjusted for age and sex. Model 2 further adjusted for SBP, DBP, smoking status, drinking status, and family history of diabetes based on Model 1. Model 3 further adjusted for TC, FPG, LDL-C, ALT, AST, BUN, and Scr based on Model 2. Values are expressed as HRs with 95% CIs. *P* for trend was calculated by treating the median value of each AIP tertile as a continuous variable. SBP, systolic blood pressure; DBP, diastolic blood pressure; BMI, body mass index; FPG, fasting plasma glucose; TC, total cholesterol; TG, triglyceride; HDL-C, high-density lipoprotein cholesterol; LDL-C, low-density lipoprotein cholesterol; ALT, alanine aminotransferase; AST, aspartate aminotransferase; BUN, blood urea nitrogen; Scr, serum creatinine; AIP, atherogenic index of plasma; HRs, hazard ratios; Cis, confidence intervals.

### BMI-stratified nonlinear associations between AIP and prediabetes risk

**Figure 4:**
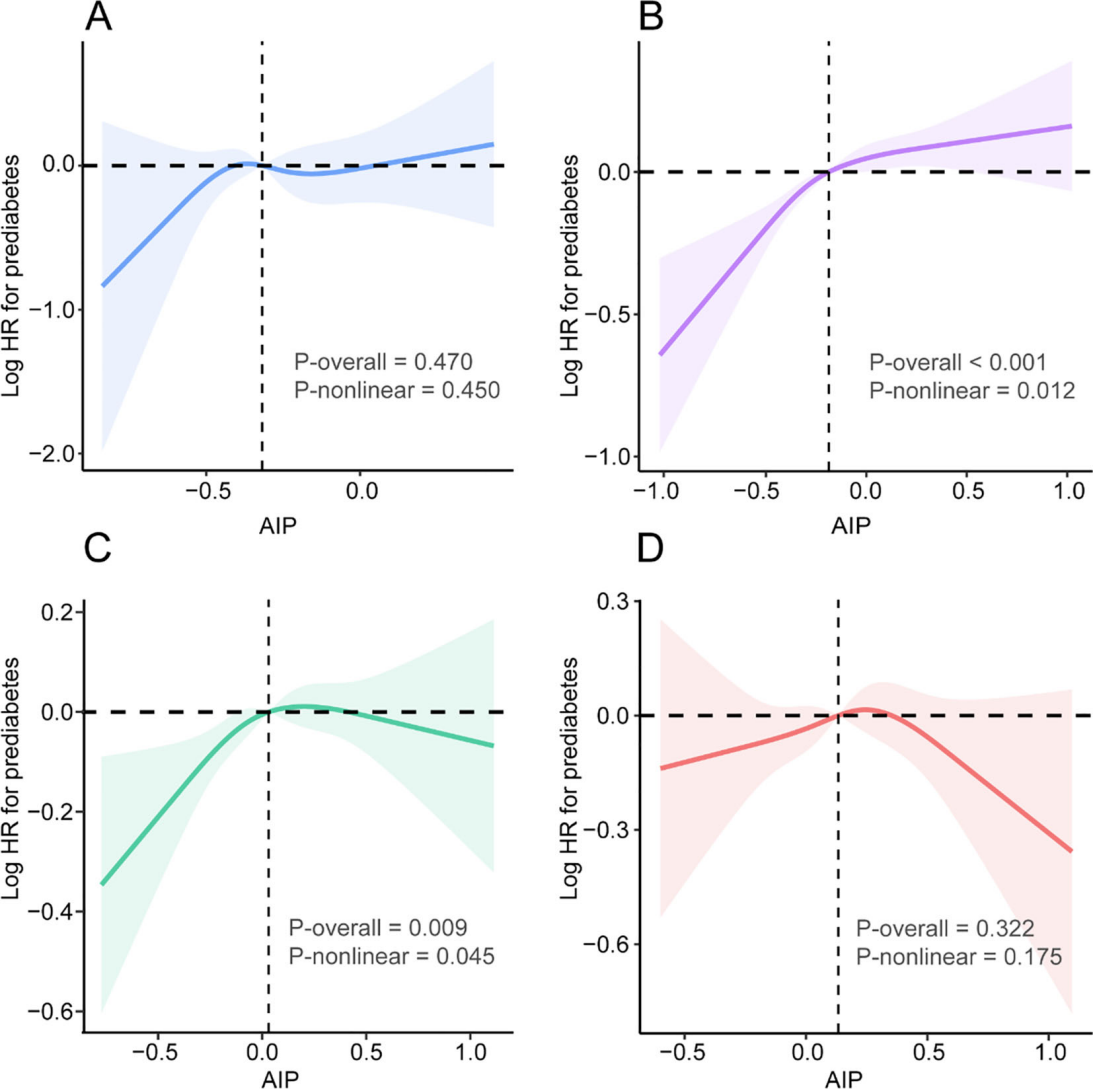
RCS analysis between AIP with prediabetes in participants with different BMI status (underweight, normal weight, overweight, and obesity). **(A)** Underweight; **(B)** Normal weight; **(C)** Overweight; **(D)** Obesity. We chose four percentiles (0.05, 0.35, 0.65, 0.95) as the knots of RCS to form a smooth curve. The shaded areas represent the 95% CI. The model was adjusted for age, sex, SBP, DBP, smoking status, drinking status, family history of diabetes, TC, FPG, LDL-C, ALT, AST, BUN, and Scr. SBP systolic blood pressure, DBP diastolic blood pressure, BMI body mass index, FPG fasting plasma glucose, TC total cholesterol, TG triglyceride, HDL-C high-density lipoprotein cholesterol, LDL-C low-density lipoprotein cholesterol, ALT alanine aminotransferase, AST aspartate aminotransferase, BUN blood urea nitrogen, Scr serum creatinine, AIP atherogenic index of plasma, RCS restricted cubic spline.

To further explore the dose-response relationship between AIP and the risk of prediabetes, RCS curves were constructed with knots at the 5th, 35th, 65th, and 95th percentiles (**Figure 4**). After adjustment for all covariates, a significant nonlinear association between AIP and prediabetes risk was observed among participants with normal weight (**Figure 4B**) and those who were overweight (**Figure 4C**). In the normal-weight group, the relationship between AIP and prediabetes risk displayed an inflection point at an AIP value of -0.20. Below this threshold, the risk of prediabetes increased sharply with rising AIP (HR = 1.94, 95% CI 1.45-2.58, *P* < 0.001), whereas above the threshold, the association became positive but much weaker (HR = 1.18, 95% CI 1.02-1.37, *P* = 0.032). The log-likelihood ratio test confirmed a significant nonlinear pattern (*P* = 0.009) (**Supplementary Table 7**). Similarly, in the overweight group, a turning point was identified at AIP = 0.057. Below this value, higher AIP was significantly associated with increased prediabetes risk (HR = 1.51, 95% CI 1.21-1.89, *P* < 0.001), while above this threshold, the risk plateaued (HR = 0.91, 95% CI 0.75-1.10, *P* = 0.328), indicating a nonlinear trend (*P* = 0.005) (**Supplementary Table 7**). In contrast, no significant nonlinear or overall associations were found in the underweight (**Figure 4A**) or obese (**Figure 4D**) groups (*P* > 0.05 for both overall and nonlinear tests).

### ROC analysis

**Figure 5:**
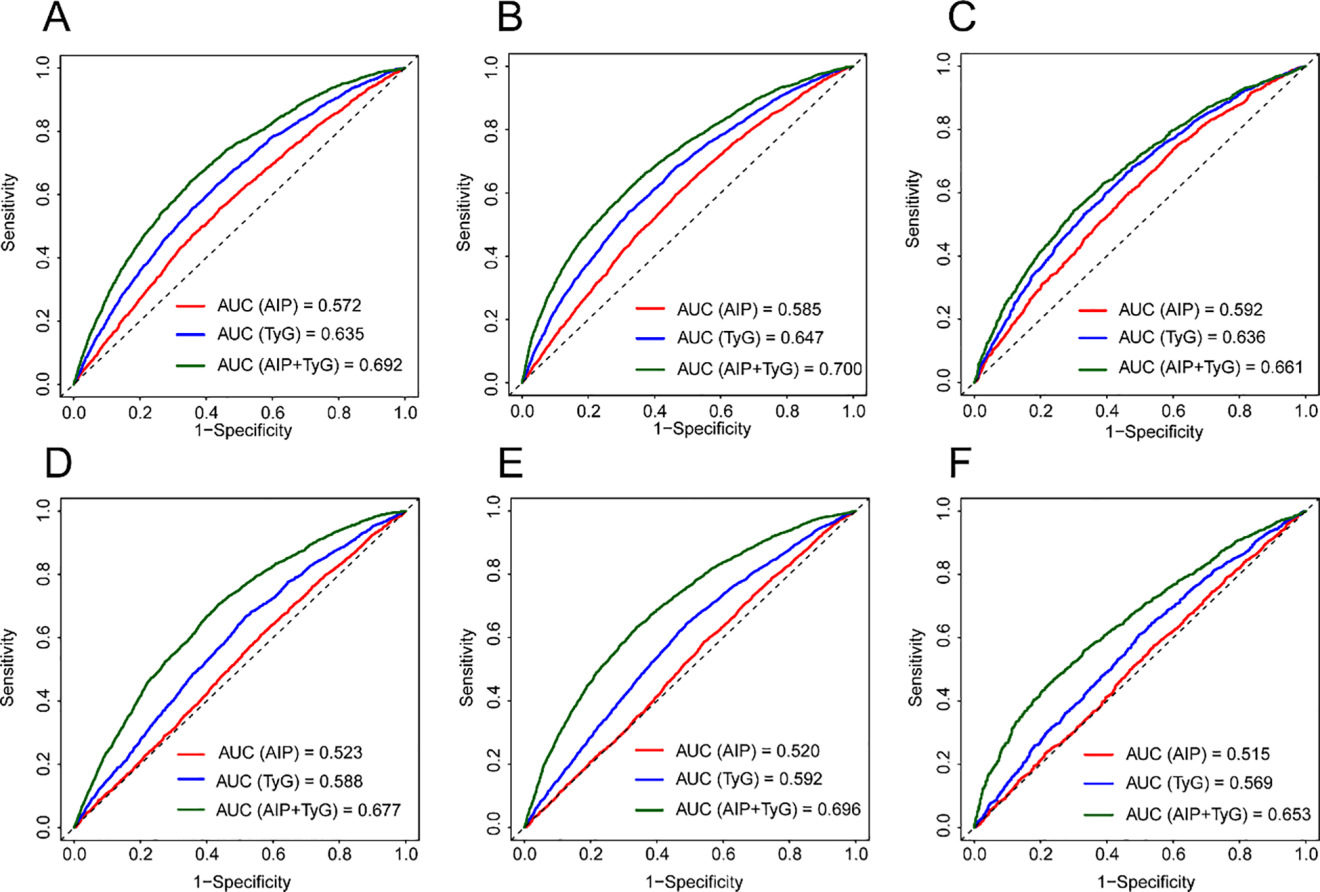
ROC curve analysis of the AIP and TyG index in predicting prediabetes. **(A)** Normal weight, 3 years; **(B)** Normal weight, 4 years; **(C)** Normal weight, 5 years; **(D)** Overweight, 3 years; **(E)** Overweight, 4 years; **(F)** Overweight, 5 years. Time-dependent ROC curve was used to construct the model, with time cutoff value of 3 years, 4 years, and 5 years. ROC, receiver operating characteristic, AIP atherogenic index of plasma, TyG index, triglyceride glucose index.

Because significant associations between AIP and prediabetes risk were observed only in the normal-weight and overweight groups, time-dependent ROC analyses were performed in these two subgroups (**Figure 5**). The TyG index showed a certain level of discriminatory ability, and the addition of AIP provided a modest but consistent improvement in predictive performance. These findings suggest that AIP may provide additional supplementary information beyond the TyG index.

### Subgroup analysis

Subgroup analyses stratified by age, sex, and family history of diabetes were performed to further examine the robustness of the association between AIP and the risk of prediabetes. Subgroup analyses showed that the relationship was significantly modified by age and sex in the normal-weight and overweight groups (**Supplementary Table 8**). The association was stronger among participants aged ≤60 years and in women, whereas it was not significant in older individuals or in men (*P* for interaction < 0.05). No significant interaction was observed with a family history of diabetes.

## Discussion

In this large cohort of Chinese adults, BMI substantially modified the association between AIP and FPG-defined prediabetes risk. This association was primarily observed in normal-weight and overweight individuals, in whom higher AIP was associated with a greater risk of prediabetes and showed a significant nonlinear pattern. By contrast, no significant association was observed in the underweight or obese groups after full adjustment. In addition, in normal-weight individuals, prediabetes risk increased more steeply below an AIP of -0.20, whereas in overweight individuals the corresponding turning point was 0.057. These findings suggest that the risk relationship between AIP and prediabetes may differ according to BMI status. Taken together, our results support BMI-specific interpretation of AIP in the assessment of FPG-defined prediabetes risk, particularly among individuals with normal weight or overweight.

Previous studies have explored the association between AIP and dysglycemia, but their findings have been inconsistent and have left several important gaps. Some studies have reported a linear association between AIP and prediabetes or diabetes risk, suggesting that higher AIP levels correspond proportionally to worsening glycemic outcomes. For example, a cross-sectional analysis of a US population found a linear association between AIP and the prevalence of prediabetes and diabetes (OR = 2.49, 95% CI: 1.75, 3.54) (21). In contrast, most prior analyses have hinted at a nonlinear pattern, suggesting that even slight elevations in AIP may be associated with disproportionately increased metabolic risk at lower ranges (22–24). For example, a cross-sectional analysis from the CHARLS study reported a threshold effect, showing that AIP was positively associated with the risk of prediabetes only when AIP exceeded 0.29 (OR = 2.24, 95% CI: 1.67-3.00). In contrast, when AIP was below 0.29, no significant association was observed (OR = 1.28, 95% CI: 0.91-1.81) (23). However, most of these analyses are based on relatively small samples or cross-sectional data lacking follow-up. Importantly, there is well-established evidence that adiposity strongly influences lipid metabolism, insulin resistance, and cardiometabolic vulnerability (25–27). A previous study also reported that the association between AIP and prediabetes differed by BMI category, with stronger effects observed among individuals with BMI <25 kg/m² (28). However, that study dichotomized BMI (<25 vs. ≥25 kg/m²) rather than applying BMI categories appropriate for Chinese adults (underweight <18.5, normal weight 18.5-23.9, overweight 24.0-27.9, and obesity ≥28 kg/m²) (16). Moreover, the study identified the interaction only through subgroup analyses and did not further characterize how the dose–response relationship varied across adiposity phenotypes. Thus, to address the above gaps, our study incorporated a much larger, community-based cohort, with a median follow-up of 3.0 years, to characterize both the association of AIP of prediabetes and the modifying role of BMI, thereby providing new insights into how adiposity modifies the impact of dyslipidemia on prediabetes risk. By integrating RCS modeling with BMI stratification tailored to Chinese population standards, our analyses revealed a significant interaction between BMI and AIP (*P* for interaction < 0.001) and identified distinct nonlinear thresholds that varied markedly by adiposity phenotype. In the normal-weight group, the AIP-prediabetes curve displayed a turning point at AIP = -0.20, below which the risk of prediabetes increased sharply, whereas above this threshold the relationship persisted but was notably weaker. In the overweight group, the inflection point shifted rightward to AIP = 0.057, with risk elevation observed only below this value and a plateau thereafter. Importantly, these inflection points should be interpreted as model-derived exploratory turning points rather than definitive clinical cut-offs. External validation in independent cohorts is needed before these values can be considered for clinical risk stratification. In addition, no significant association between AIP and prediabetes was observed in underweight or obese individuals. These heterogeneities indicate that metabolic vulnerability related to AIP manifests at substantially different levels of lipid atherogenicity depending on BMI. Our findings complement and extend earlier studies that reported nonlinear associations between AIP and dysglycemia (22–24) and refine observations from a previous analysis showing stronger AIP effects among individuals with BMI <25 kg/m² (28). The restriction of the nonlinear AIP-prediabetes association to normal-weight and overweight categories implies that early dyslipidemia may disproportionately accelerate glycemic deterioration in individuals who have not yet developed overt obesity. From an epidemiological perspective, these BMI-specific nonlinear patterns may inform future research on BMI-specific interpretation of AIP for FPG-defined prediabetes risk. However, given residual confounding and the lack of HbA1c, OGTT, medication, lifestyle, and socioeconomic data, these findings should not be interpreted as establishing a clinical risk stratification strategy.

These BMI-specific nonlinear patterns imply that adiposity plays a role in shaping the relationship between dyslipidemia and glycemic deterioration. Normal-weight individuals often exhibit lower metabolic reserve and greater susceptibility to early lipid disturbances, such that even modest increases in AIP may disproportionately impair insulin sensitivity. This aligns with the “metabolically unhealthy normal-weight” phenotype described in prior literature (29–31). By contrast, overweight individuals typically have a higher baseline lipid burden and stronger early-stage compensatory insulin secretion (32–34). Therefore, a greater degree of lipid atherogenicity is required before additional glycemic deterioration becomes detectable, resulting in a right-shifted threshold (normal-weight AIP = -0.20 → overweight AIP = 0.057). In underweight and obese individuals, no significant association between AIP and prediabetes risk was observed in the fully adjusted analyses. These null findings should be interpreted cautiously. On the one hand, they may reflect genuine biological heterogeneity across BMI phenotypes, as adiposity is known to influence lipid metabolism, insulin resistance, and cardiometabolic vulnerability (25–27). In addition, prior studies suggest that the dominant pathways leading to dysglycemia may differ between individuals with low body weight and those with obesity (35–37), On the other hand, the relatively smaller sample sizes in the underweight and obese groups in our study may have limited statistical power to detect modest associations. Therefore, our findings do not exclude a possible association in these strata, and the proposed biological explanations should be considered hypothesis-generating rather than definitive.

This longitudinal study of more than 100,000 Chinese adults, with BMI stratification based on Chinese population standards, provides important insight into how adiposity modifies the association between AIP and incident prediabetes. However, several limitations should be acknowledged. First, a substantial number of participants were excluded because of missing AIP measurements. Because AIP was the primary exposure, missing AIP values were not imputed to avoid model-dependent exposure misclassification. However, compared with included participants, excluded participants generally had a less favorable metabolic profile, which may limit the representativeness of the analytic cohort. Therefore, selection bias related to missing AIP cannot be ruled out, and the observed associations may have been biased. Second, the cohort consisted of individuals undergoing health examinations at a single center, which may limit the external generalizability of our findings. Third, the present study assessed only FPG-defined incident prediabetes because HbA1c and OGTT data were unavailable. Thus, the outcome should not be interpreted as prediabetes defined by the full ADA criteria. This may have introduced misclassification and underascertainment, because isolated HbA1c-defined prediabetes, impaired glucose tolerance, and abnormal baseline HbA1c/OGTT despite normal FPG could not be identified. Consequently, the incidence of prediabetes may have been underestimated, and the observed associations may have been biased, most likely toward attenuation. In addition, excluding participants who progressed directly to diabetes may have limited our ability to assess broader glucose metabolism deterioration. Fourth, AIP and covariates were measured only at baseline; therefore, temporal changes in lipid profiles or BMI could not be evaluated. Fifth, information on diet, physical activity, socioeconomic factors, inflammatory markers, and medication use was not available in the source dataset and therefore could not be adjusted for. These factors may influence TG and HDL-C levels, AIP values, and glucose metabolism. In particular, lipid-lowering medications such as statins or fibrates may alter TG and HDL-C levels, while other medications may affect glycemic status. Because medication use and lifestyle factors are often related to underlying cardiometabolic risk, residual confounding cannot be excluded. Therefore, the observed associations should be interpreted cautiously and should not be considered sufficient to establish clinical risk stratification without further validation. Sixth, insulin levels were not measured, precluding assessment of insulin resistance using standard indices such as the homeostasis model assessment of insulin resistance (HOMA-IR). Finally, although interactions and nonlinearities were explored across BMI categories, the relatively small sample sizes in the underweight and obese groups may have reduced statistical power to detect associations in these strata. Therefore, the absence of statistically significant associations in these groups should not be interpreted as definitive evidence of no biological relationship.

## Conclusion

In this large cohort of Chinese adults, BMI modified the association between AIP and FPG-defined prediabetes risk, with significant nonlinear associations observed only in normal-weight and overweight individuals. The identified inflection points are exploratory and should not be interpreted as definitive clinical cut-offs. External validation is needed before clinical risk-stratification use.

## Data Availability

Publicly available datasets were analyzed in this study. Dryad repositories (https://datadryad.org/stash) contain the datasets used in this investigation.
