# Supplementary material for: BMI-specific nonlinear associations and threshold effects of the atherogenic index of plasma on incident prediabetes: insights from 100473 Chinese adults
Source: Front Endocrinol (Lausanne). 2026 May 14;17:1813692. doi: 10.3389/fendo.2026.1813692 (PMC13218080; doi:10.3389/fendo.2026.1813692)
Supplement: Supplementary file 1 [file DataSheet1.docx]

**Supplementary Table 1.** The percentage of missing values ​​for excluded variables

| Variables | Missing number | Missing Proportion, % |
| --- | --- | --- |
| SBP | 13 | 0.013 |
| DBP | 13 | 0.013 |
| TC | 1 | 0.001 |
| LDL | 162 | 0.161 |
| ALT | 372 | 0.370 |
| AST | 58,355 | 58.080 |
| BUN | 2,307 | 2.296 |
| Scr | 1,216 | 1.210 |
| Smoking status | 72,789 | 72.446 |
| Drinking status | 72,789 | 72.446 |

Abbreviations: SBP systolic blood pressure, DBP diastolic blood pressure, TC total cholesterol, LDL-C low-density lipoprotein cholesterol, ALT alanine aminotransferase, AST aspartate aminotransferase, BUN blood urea nitrogen, Scr serum creatinine.

**Supplementary Table 2.** Comparison of missing value characteristics before and after multiple imputation.

| **Variables** | **Before Interpolation (n=100473)** | **After Interpolation (n=100473)** |
| --- | --- | --- |
| SBP, mmHg | 118.08 ± 16.08 | 118.08 ± 16.08 |
| DBP, mmHg | 73.75 ± 10.77 | 73.76 ± 10.77 |
| Smoking status, % |  |  |
| Current | 19.36 | 18.10 |
| Once | 3.94 | 3.77 |
| Never | 76.69 | 78.13 |
| Drinking status, % |  |  |
| Current | 2.33 | 2.14 |
| Once | 16.46 | 15.40 |
| Never | 81.21 | 82.46 |
| TC, mmol/L | 4.75 ± 0.88 | 4.75 ± 0.88 |
| LDL-C, mmol/L | 2.74 ± 0.67 | 2.74 ± 0.67 |
| ALT, U/L | 23.09 ± 21.47 | 23.09 ± 21.47 |
| AST, U/L | 23.71 ± 12.71 | 23.71 ± 12.59 |
| BUN, mmol/L | 4.63 ± 1.16 | 4.63 ± 1.16 |
| Scr, umol/L | 69.90 ± 15.72 | 69.90 ± 15.72 |

Abbreviations: SBP systolic blood pressure, DBP diastolic blood pressure, TC total cholesterol, LDL-C low-density lipoprotein cholesterol, ALT alanine aminotransferase, AST aspartate aminotransferase, BUN blood urea nitrogen, Scr serum creatinine.

**Supplementary Table 3.** Collinearity analysis.

| Variables | GVIF | Df | GVIF^(1/(2*Df)) |
| --- | --- | --- | --- |
| Age | 1.29 | 1 | 1.14 |
| Sex | 1.95 | 1 | 1.40 |
| SBP | 2.07 | 1 | 1.44 |
| DBP | 1.95 | 1 | 1.40 |
| Smoking status | 1.15 | 2 | 1.04 |
| Drinking status | 1.11 | 2 | 1.03 |
| Family history of diabetes | 1.01 | 1 | 1.00 |
| TC | 4.61 | 1 | 2.15 |
| FPG | 1.01 | 1 | 1.01 |
| LDL | 4.38 | 1 | 2.09 |
| ALT | 3.63 | 1 | 1.90 |
| AST | 3.43 | 1 | 1.85 |
| BUN | 1.19 | 1 | 1.09 |
| Scr | 1.92 | 1 | 1.38 |

Abbreviations: SBP systolic blood pressure, DBP diastolic blood pressure, FPG fasting plasma glucose, TC total cholesterol, LDL-C low-density lipoprotein cholesterol, ALT alanine aminotransferase, AST aspartate aminotransferase, Scr serum creatinine, BUN blood urea nitrogen.

**Supplementary Table 4.** Baseline characteristics of participants with and without prediabetes.

| **Variables** | **Overall (n=100473)** | **Non-prediabetes (n=88102)** | **Prediabetes (n=12371)** | ***P*** |
| --- | --- | --- | --- | --- |
| Age, years | 42.91 ± 12.46 | 42.06 ± 12.04 | 49.01 ± 13.58 | <0.001 |
| Sex, n (%) |  |  |  | <0.001 |
| Male | 52217(51.97) | 44469(50.47) | 7748(62.63) |  |
| Female | 48256(48.03) | 43633(49.53) | 4623(37.37) |  |
| SBP, mmHg | 118.08 ± 16.08 | 117.08 ± 15.62 | 125.17 ± 17.51 | <0.001 |
| DBP, mmHg | 73.76 ± 10.77 | 73.21 ± 10.57 | 77.61 ± 11.38 | <0.001 |
| BMI | 23.09 ± 3.22 | 22.91 ± 3.17 | 24.38 ± 3.25 | <0.001 |
| Smoking status, n (%) | |  |  | <0.001 |
| Current | 18188(18.10) | 15512(17.61) | 2676(21.63) |  |
| Once | 3790(3.77) | 3276(3.72) | 514(4.15) |  |
| Never | 78495(78.13) | 69314(78.67) | 9181(74.21) |  |
| Drinking status, n (%) | |  |  | <0.001 |
| Current | 2152(2.14) | 1830(2.08) | 322(2.60) |  |
| Once | 15469(15.40) | 13255(15.05) | 2214(17.90) |  |
| Never | 82852(82.46) | 73017(82.88) | 9835(79.50) |  |
| Family history of diabetes, n (%) | |  |  | 0.02 |
| No | 98261(97.80) | 86200(97.84) | 12061(97.49) |  |
| Yes | 2212(2.20) | 1902(2.16) | 310(2.51) |  |
| FPG, mmol/L | 4.79 ± 0.47 | 4.75 ± 0.47 | 5.03 ± 0.40 | <0.001 |
| TC, mmol/L | 4.75 ± 0.88 | 4.72 ± 0.88 | 4.92 ± 0.90 | <0.001 |
| TG, mmol/L | 1.06(0.74,1.58) | 1.02(0.72,1.52) | 1.30(0.90,1.94) | <0.001 |
| HDL-C, mmol/L | 1.38 ± 0.30 | 1.39 ± 0.31 | 1.34 ± 0.29 | <0.001 |
| LDL-C, mmol/L | 2.74 ± 0.67 | 2.73 ± 0.67 | 2.84 ± 0.67 | <0.001 |
| ALT, U/L | 17.70(12.80,26.60) | 17.10(12.40,26.00) | 21.00(14.70,31.00) | <0.001 |
| AST, U/L | 22.00(17.60,27.96) | 22.00(17.40,27.64) | 23.43(19.00,29.55) | <0.001 |
| BUN, mmol/L | 4.63 ± 1.16 | 4.61 ± 1.15 | 4.84 ± 1.16 | <0.001 |
| Scr, umol/L | 69.90 ± 15.72 | 69.44 ± 15.67 | 73.15 ± 15.63 | <0.001 |
| AIP | -0.11(-0.30,0.10) | -0.12(-0.31,0.08) | -0.003(-0.20,0.20) | <0.001 |

Abbreviations: SBP systolic blood pressure, DBP diastolic blood pressure, BMI body mass index, FPG fasting plasma glucose, TC total cholesterol, TG triglyceride, HDL-C high-density lipoprotein cholesterol, LDL-C low-density lipoprotein cholesterol, ALT alanine aminotransferase, AST aspartate aminotransferase, BUN blood urea nitrogen, Scr serum creatinine, AIP atherogenic index of plasma.

**Supplementary Table 5.** Baseline characteristics of included and excluded participants.

| **Variables** | **Exclude (n=111360)** | **Include (n=100473)** | ***P*** |
| --- | --- | --- | --- |
| Age, years | 41.36 ± 12.78 | 42.91 ± 12.46 | <0.001 |
| Sex, n (%) |  |  | <0.001 |
| Male | 63906(57.39) | 52217(51.97) |  |
| Female | 47454(42.61) | 48256(48.03) |  |
| SBP, mmHg | 119.95 ± 16.59 | 118.08 ± 16.08 | <0.001 |
| DBP, mmHg | 74.56 ± 10.84 | 73.76 ± 10.77 | <0.001 |
| BMI | 23.36 ± 3.44 | 23.09 ± 3.22 | <0.001 |
| Smoking status, n (%) |  |  | <0.001 |
| Current | 6714(20.63) | 18188(18.10) |  |
| Once | 1467(4.51) | 3790(3.77) |  |
| Never | 24365(74.86) | 78495(78.13) |  |
| Drinking status, n (%) |  |  | <0.001 |
| Current | 706(2.17) | 2152(2.14) |  |
| Once | 4398(13.51) | 15469(15.40) |  |
| Never | 27442(84.32) | 82852(82.46) |  |
| Family history of diabetes, n (%) |  |  | <0.001 |
| No | 109228(98.09) | 98261(97.80) |  |
| Yes | 2132(1.91) | 2212(2.20) |  |
| FPG, mmol/L | 5.03 ± 0.70 | 4.79 ± 0.47 | <0.001 |
| TC, mmol/L | 4.67 ± 0.92 | 4.75 ± 0.88 | <0.001 |
| TG, mmol/L | 1.37 ± 1.10 | 1.06(0.74,1.58) | <0.001 |
| HDL-C, mmol/L | 1.32 ± 0.32 | 1.38 ± 0.30 | <0.001 |
| LDL-C, mmol/L | 2.90 ± 0.73 | 2.74 ± 0.67 | <0.001 |
| ALT, U/L | 24.74 ± 22.68 | 17.70(12.80,26.60) | <0.001 |
| AST, U/L | 24.43 ± 12.03 | 22.00(17.60,27.96) | <0.001 |
| BUN, mmol/L | 4.68 ± 1.22 | 4.63 ± 1.16 | <0.001 |
| Scr, umol/L | 70.22 ± 15.88 | 69.90 ± 15.72 | <0.001 |
| AIP | 0.06 ± 0.31 | -0.11(-0.30,0.10) | <0.001 |

Abbreviations: SBP systolic blood pressure, DBP diastolic blood pressure, BMI body mass index, FPG fasting plasma glucose, TC total cholesterol, TG triglyceride, HDL-C high-density lipoprotein cholesterol, LDL-C low-density lipoprotein cholesterol, ALT alanine aminotransferase, AST aspartate aminotransferase, BUN blood urea nitrogen, Scr serum creatinine, AIP atherogenic index of plasma.

**Supplementary Table 6.** Association between AIP and risk of prediabetes in the overall population.

| **Groups** | **Model 1** | | **Model 2** | | **Model 3** | |
| --- | --- | --- | --- | --- | --- | --- |
|  | **HR (95%CI)** | ***P*** | **HR (95%CI)** | ***P*** | **HR (95%CI)** | ***P*** |
| Underweight |  |  |  |  |  |  |
| AIP (Continuous) | 1.87(1.76,1.99) | <0.001 | 1.31(1.23,1.40) | <0.001 | 1.19(1.11,1.27) | <0.001 |
| The tertiles of AIP | |  |  |  |  |  |
| Q1 | Reference |  | Reference |  | Reference |  |
| Q2 | 1.32(1.25,1.39) | <0.001 | 1.27(1.20,1.33) | <0.001 | 1.18(1.12,1.24) | <0.001 |
| Q3 | 1.62(1.54,1.70) | <0.001 | 1.49(1.42,1.57) | <0.001 | 1.29(1.23,1.36) | <0.001 |
| *P* for trend |  | <0.001 |  | <0.001 |  | <0.001 |

Model 1 adjusted for age and sex. Model 2 further adjusted for SBP, DBP, smoking status, drinking status, and family history of diabetes based on Model 1. Model 3 further adjusted for TC, FPG, LDL-C, ALT, AST, BUN, and Scr based on Model 2. Values are expressed as HRs with 95% CIs. *P* for trend was calculated by treating the median value of each AIP tertile as a continuous variable.

Abbreviations: SBP systolic blood pressure, DBP diastolic blood pressure, FPG fasting plasma glucose, TC total cholesterol, TG triglyceride, HDL-C high-density lipoprotein cholesterol, LDL-C low-density lipoprotein cholesterol, ALT alanine aminotransferase, AST aspartate aminotransferase, BUN blood urea nitrogen, Scr serum creatinine, AIP atherogenic index of plasma, HRs hazard ratios, Cis, confidence intervals.


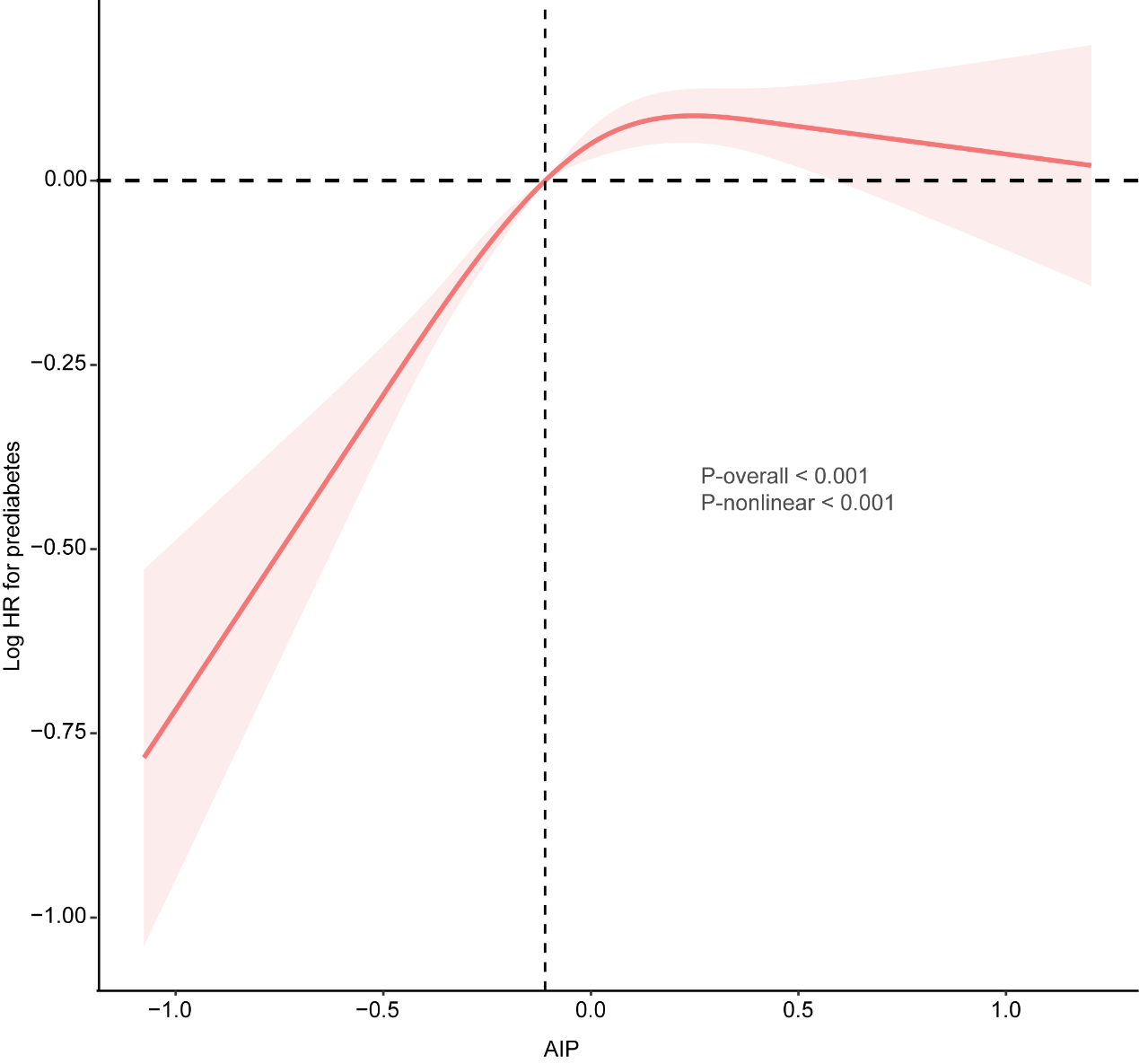


**Supplementary Figure 1.** RCS analysis between AIP with prediabetes in the overall population. We chose four percentiles (0.05, 0.35, 0.65, 0.95) as the knots of RCS to form a smooth curve. The shaded areas represent the 95% CI. The model was adjusted for age, sex, SBP, DBP, smoking status, drinking status, family history of diabetes, TC, FPG, LDL-C, ALT, AST, BUN, and Scr. Abbreviations: SBP systolic blood pressure, DBP diastolic blood pressure, BMI body mass index, FPG fasting plasma glucose, TC total cholesterol, TG triglyceride, HDL-C high-density lipoprotein cholesterol, LDL-C low-density lipoprotein cholesterol, ALT alanine aminotransferase, AST aspartate aminotransferase, BUN blood urea nitrogen, Scr serum creatinine, AIP atherogenic index of plasma, RCS restricted cubic spline.

**Supplementary Table 7**. The result of the two-piecewise Cox regression model.

| Groups | Turning point (K) | <K segment effect 1 | >K segment effect 2 | *P* for log-likelihood ratio test |
| --- | --- | --- | --- | --- |
| Normal weight | -0.20 | 1.94(1.45-2.58), *P* < 0.001 | 1.18(1.02-1.37), *P* = 0.032 | 0.009 |
|  |  |  |  |  |
| Overweight | 0.057 | 1.51 (1.21-1.89), *P* < 0.001 | 0.91 (0.75-1.10), *P* = 0.328 | 0.005 |
|  |  |  |  |  |

**Supplementary Table 8.** Subgroup analyses according to age (≤60/>60 years), sex, and Family history of diabetes.

| **Groups** | **HR (95% CI)** | ***P*** | ***P* for interaction** |
| --- | --- | --- | --- |
| **Underweight** |  |  |  |
| Age, years |  |  | 0.35 |
| ≤60 | 1.03(0.82,1.29) | 0.81 |  |
| >60 | 0.86(0.55,1.36) | 0.53 |  |
| Sex |  |  | 0.92 |
| Male | 0.97(0.77,1.21) | 0.77 |  |
| Female | 1.09(0.70,1.69) | 0.71 |  |
| Family history of diabetes |  |  | 0.99 |
| No | 0.99(0.81,1.22) | 0.93 |  |
| Yes | 1.04(0.30,3.61) | 0.95 |  |
| **Normal weight** |  |  |  |
| Age, years |  |  | 0.004 |
| ≤60 | 1.36(1.21,1.54) | <0.0001 |  |
| >60 | 1(0.79,1.26) | 0.98 |  |
| Sex |  |  | < 0.0001 |
| Male | 1.14(0.99,1.32) | 0.07 |  |
| Female | 1.50(1.28,1.76) | <0.0001 |  |
| Family history of diabetes |  |  | 0.26 |
| No | 1.34(1.21,1.50) | <0.0001 |  |
| Yes | 2.24(1.16,4.32) | 0.02 |  |
| **Overweight** |  |  |  |
| Age, years |  |  | 0.01 |
| ≤60 | 1.17(1.04,1.33) | 0.01 |  |
| >60 | 1.00(0.80,1.25) | 1.00 |  |
| Sex |  |  | 0.02 |
| Male | 1.05(0.92,1.19) | 0.50 |  |
| Female | 1.35(1.10,1.64) | 0.004 |  |
| Family history of diabetes |  |  | 0.97 |
| No | 1.15(1.03,1.28) | 0.01 |  |
| Yes | 0.98(0.47,2.04) | 0.96 |  |
| **Obesity** |  |  |  |
| Age, years |  |  | 0.35 |
| ≤60 | 1.03(0.82,1.29) | 0.81 |  |
| >60 | 0.86(0.55,1.36) | 0.53 |  |
| Sex |  |  | 0.92 |
| Male | 0.97(0.77,1.21) | 0.77 |  |
| Female | 1.09(0.70,1.69) | 0.71 |  |
| Family history of diabetes |  |  | 0.99 |
| No | 0.99(0.81,1.22) | 0.93 |  |
| Yes | 1.04(0.30,3.61) | 0.95 |  |

Age, sex, SBP, DBP, smoking status, drinking status, family history of diabetes, TC, FPG, LDL-C, ALT, AST, BUN, and Scr were adjusted (Subgroup variables are not included during adjustment).

Abbreviations: SBP systolic blood pressure, DBP diastolic blood pressure, FPG fasting plasma glucose, TC total cholesterol, TG triglyceride, HDL-C high-density lipoprotein cholesterol, LDL-C low-density lipoprotein cholesterol, ALT alanine aminotransferase, AST aspartate aminotransferase, BUN blood urea nitrogen, Scr serum creatinine, HRs hazard ratios, CIs, confidence intervals.
